# Extracellular Matrix-Oriented Proteomic Analysis of Periodontal Ligament Under Mechanical Stress

**DOI:** 10.3389/fphys.2022.899699

**Published:** 2022-05-20

**Authors:** Lay Thant, Masaru Kaku, Yoshito Kakihara, Masaru Mizukoshi, Megumi Kitami, Moe Arai, Kohei Kitami, Daiki Kobayashi, Yutaka Yoshida, Takeyasu Maeda, Isao Saito, Katsumi Uoshima, Makio Saeki

**Affiliations:** ^1^ Division of Dental Pharmacology, Faculty of Dentistry & Graduate School of Medical and Dental Sciences, Niigata University, Niigata, Japan; ^2^ Division of Orthodontics, Faculty of Dentistry & Graduate School of Medical and Dental Sciences, Niigata University, Niigata, Japan; ^3^ Center for Advanced Oral Science, Faculty of Dentistry & Graduate School of Medical and Dental Sciences, Niigata University, Niigata, Japan; ^4^ Division of Bio-prosthodontics, Faculty of Dentistry & Graduate School of Medical and Dental Sciences, Niigata University, Niigata, Japan; ^5^ Omics Unit, Graduate School of Medical and Dental Sciences, Niigata University, Niigata, Japan; ^6^ Department of Structural Pathology, Kidney Research Center, Graduate School of Medical and Dental Sciences, Niigata University, Niigata, Japan

**Keywords:** periodontal ligament, extracellular matrix, collagen, matrisome, mechanical stress, proteomics, bioinformatics

## Abstract

The periodontal ligament (PDL) is a specialized connective tissue that provides structural support to the tooth and is crucial for oral functions. The mechanical properties of the PDL are mainly derived from the tissue-specific composition and structural characteristics of the extracellular matrix (ECM). The ECM also plays key roles in determining cell fate in the cellular microenvironment thus crucial in the PDL tissue homeostasis. In the present study, we determined the comprehensive ECM profile of mouse molar PDL using laser microdissection and mass spectrometry-based proteomic analysis with ECM-oriented data curation. Additionally, we evaluated changes in the ECM proteome under mechanical loading using a mouse orthodontic tooth movement (OTM) model and analyzed potential regulatory networks using a bioinformatics approach. Proteomic changes were evaluated in reference to the novel second harmonic generation (SHG)-based fiber characterization. Our ECM-oriented proteomics approach succeeded in illustrating the comprehensive ECM profile of the mouse molar PDL. We revealed the presence of type II collagen in PDL, possibly associated with the load-bearing function upon occlusal force. Mechanical loading induced unique architectural changes in collagen fibers along with dynamic compositional changes in the matrisome profile, particularly involving ECM glycoproteins and matrisome-associated proteins. We identified several unique matrisome proteins which responded to the different modes of mechanical loading in PDL. Notably, the proportion of type VI collagen significantly increased at the mesial side, contributing to collagen fibrogenesis. On the other hand, type XII collagen increased at the PDL-cementum boundary of the distal side. Furthermore, a multifaceted bioinformatics approach illustrated the potential molecular cues, including PDGF signaling, that maintain ECM homeostasis under mechanical loading. Our findings provide fundamental insights into the molecular network underlying ECM homeostasis in PDL, which is vital for clinical diagnosis and development of biomimetic tissue-regeneration strategies.

## Introduction

The extracellular matrix (ECM) is a principal tissue component that primarily provides a structural framework for tissues and plays key roles in determining cell fate in the cellular microenvironment (Frantz et al., 2010). Physical cues, such as externally applied deformation forces, biomaterial-induced forces, and cell-induced forces, evoke various cellular reactions that determine cell fate, with these broadly modified by the ECM (Kumar et al., 2017). Tight ECM-cell communication provides crucial biochemical and biomechanical cues that regulate cell behaviors; therefore, any physiological changes in the tissue can be traced back to the ECM (Iozzo and Gubbiotti, 2018). Hence, a dysfunctional ECM and its associated regulators affect tissue homeostasis, leading to a wide spectrum of pathological conditions, including skeletal disease, fibrosis, and cancer (Bonnans et al., 2014).

Proteomics involves the identification and quantification of the entire protein complement of a cell, tissue, or organism (Wilkins et al., 1996). Laser microdissection (LMD) combined with mass spectrometry-based proteomics such as liquid chromatography-tandem mass spectrometry (LC-MS/MS) enables the characterization of the protein composition of anatomically complex tissues in a sensitive and unbiased manner (Aebersold and Mann, 2016). Although the MS-based method offers the acquisition of comprehensive proteome, characterization of the ECM has been technically challenging due to the lack of basic knowledge of their protein constituents (Krasny and Huang, 2021). To overcome such difficulties, matrisome, a consensus catalog of proteins that are either part of or are associated with the ECM, was developed based on a domain-based in silico prediction algorithm (Naba et al., 2012). The matrisome comprised of two classes: the core matrisome which includes collagens, proteoglycans, and ECM glycoproteins; matrisome-associated proteins encompassing ECM-affiliated proteins, ECM regulators, and secreted factors (Naba et al., 2012; Naba et al., 2016).

The periodontal ligament (PDL) is a highly specialized fibrous connective tissue that anchors the tooth to the alveolar bone socket and is important for oral function, including the dissipation of masticatory force, neuronal feedback, and tooth eruption (Beertsen et al., 1997). The major ECM components of the PDL are type I and III collagens, with minor collagens (e.g., types V, VI, and XII), proteoglycans, and ECM glycoproteins (Butler et al., 1975; Beertsen et al., 1997; Nanci and Bosshardt, 2006). Mechanoregulation of the ECM in the PDL is of clinical interest because controlling PDL tissue homeostasis under mechanical loading is crucial in dental practice, such as in occlusal adjustment and managing orthodontic tooth movement (OTM). Moreover, the PDL is an adequate *in vivo* experimental model for studying ECM organization and remodeling under mechanical loading (Kaku and Yamauchi, 2014) because of its well-organized fiber alignment, rapid tissue-turnover rate (Sodek, 1977), good responsiveness, and ease of controlling the mode of mechanical loading (Nozaki et al., 2010; Kaku et al., 2016; Mizukoshi et al., 2021).

Although previous studies have attempted to identify the unique genes/proteins in the PDL (Nishida et al., 2007; Yamada et al., 2007) the global landscape of the ECM proteome in the PDL, even in the physiological state, remains unclear. Additionally, understanding changes in the ECM proteome under mechanical loading may accelerate its etiological consideration in various clinical conditions. Thus, studying the detailed ECM proteome of the PDL may enable a more precise diagnosis of the clinical state and its variations in individuals (Taha and Naba, 2019). Moreover, understanding the ECM proteome is indispensable for developing biomimetic tissue-regeneration protocols (Fernandes and Yang, 2016).

In this study, we analyzed the ECM proteome of the mouse molar PDL using LMD and LC-MS/MS with ECM-oriented data curation. Additionally, we evaluated changes in the ECM proteome under mechanical loading using a mouse OTM model and analyzed potential regulatory networks using bioinformatics approaches. Proteomic changes were evaluated in reference to the novel second harmonic generation (SHG)-based fiber characterization to better illustrate the homeostatic balance of the ECM network under different modes of mechanical loading.

## Methods

### Ethics Statement

The Niigata University Animal Experiment Ethics Committee reviewed and approved all animal procedures (SA00532). All animal handling and experiments strictly followed the ARRIVE guidelines for animal research reporting for *in vivo* experiments.

### Mouse Molar Orthodontic Tooth Movement

Eight-week-old male mice (C57BL/6J) were obtained from Charles River Laboratories Japan (Yokohama, Japan) and maintained in the animal facility of Niigata University. Mice were anesthetized with intraperitoneal injections of chloral hydrate (400 mg/kg body weight). Ni-Ti closed-coil springs (25 gf; TOMY International Inc., Tokyo, Japan) were inserted between the left maxillary 1^st^ molars and incisors to induce mesial movement of the 1^st^ molars (Mizukoshi et al., 2021). The ends of the Ni-Ti springs were tied to the molars and incisors with stainless steel wire (φ 0.09 mm) and secured with light-cure composite resin (BEAUTIFIL Flow; Shofu, Kyoto, Japan). At 14 days after coil application, the mice were sacrificed, and both sides of the maxilla were harvested and fixed with 4% w/v paraformaldehyde (PFA) for 24 h. The contralateral 1^st^ molars served as the controls.

### Micro-Computed Tomography

Micro-CT images of the maxilla were obtained with a CosmoScan GX high-resolution X-ray tomographic system (Rigaku Corporation, Tokyo, Japan) at 90 kV and 88-μA irradiation and structurally examined using Analyze software (v.12.0; AnalyzeDirect, Stilwell, KS, United States). The distance between the closest points of the 1^st^ and 2^nd^ molars was measured after the outline of the teeth was defined with a threshold intensity of between 6,000 and 12,000 Hounsfield units (*n* = 5) (Mizukoshi et al., 2021).

### Sample Preparation and Histology

Upper 1^st^ molars with surrounding tissue were dissected, decalcified with 10% (w/v) EDTA for 3 weeks at 4°C, and embedded in Tissue-Tek O.C.T. compound (Sakura Finetek, Tokyo, Japan) or paraffin according to a standard protocol (Kaku et al., 2016; Mizukoshi et al., 2021). Sagittal cryosections (10-µm thick) were prepared on membrane-coated slides (Leica Biosystems, Wetzlar, Germany), and 5-µm thick sagittal paraffin sections were prepared using a microtome (REM-710+MC-802C; Yamato Kohki, Saitama, Japan). Paraffin sections were stained with hematoxylin and eosin and a tartrate-resistant acid phosphatase/alkaline phosphatase (TRAP/ALP)-staining kit (FUJIFILM Wako Pure Chemical Corporation, Osaka, Japan) according to the manufacturer’s instructions.

### Second Harmonic Generation Microscopy and Individual Fiber Analysis

Images were developed using a custom-built multiphoton microscope at Niigata University (Zeiss, Oberkochen, Germany). The second harmonic generation (SHG) signal was generated by a tunable Ti-sapphire laser with an excitation wavelength of 800 nm. Morphological analysis of the SHG images was performed using CT-FIRE software with default settings (Bredfeldt et al., 2014) to measure the individual collagen-fiber metrics of number, length, width, and angulation. The parameters were expressed in pixels.

### Laser Microdissection

Cryosections were stained with 0.05% (w/v) Toluidine Blue (Merck), and PDL samples from the mesial and distal sides of the distal root PDL were separately dissected using an LMD 7000 microdissection system (Leica, Wetzlar, Germany) with a cutting speed of 10 mm/s. The LMD settings were optimized for capture under a 10 × objective lens with a laser power of 20 mW. Approximately 20 cryosections were prepared for each mouse, and a total of eight mice (∼ 160 cryosections) were used to obtain enough protein for proteomic analysis. The proteomic experiment was repeated three times (total of 24 mice) for getting data from three biological replicates (*n* = 3). Samples were kept at −80°C for subsequent analysis.

### Peptide-Sample Preparation

Micro-dissected tissue samples were dispersed and equilibrated with 50 µL of SDS extraction buffer [2% (w/v) SDS, 50 mM Tris-HCl (pH 8.8), 2% (v/v) 2-mercaptoethanol, and 10% (v/v) glycerol] for 30 min, followed by incubation at 95°C for 20 min and 60°C for 2 h. The mixtures were centrifuged at 10,000 × g for 5 min to obtain the supernatants as protein extracts. The extracts were co-polymerized with acrylamide (Lu and Zhu, 2005) and in-gel digested with trypsin (T6567; Sigma-Aldrich, St. Louis, MO, United States) to generate dithiothreitol-reduced iodoacetamide-alkylated tryptic peptides (Katayama et al., 2001).

### Lipid Chromatography-Tandem Mass Spectrometry (LC-MS/MS)

Trypsin-digested peptides were dissolved in 0.3% formic acid and filtered through a 0.45-µm Ultrafree-MC membrane filter (Merck-Millipore, Billerica, MA, United States). Peptide concentration of samples was estimated using a modified bicinchoninic acid assay (Kappor et al., 2009). Samples containing 0.13 µg peptide were analyzed in triplicate using direct-injection mode on an Eksigent NanoLC 415 nano-flow liquid chromatography system (Sciex, Framingham, MA, United States) using a 75 μm × 150 mm C18 spray-tip column (3 μm, 120 Å; Nikkyo Technos, Tokyo, Japan) coupled with a TripleTOF 5600 + tandem mass spectrometer (Sciex). Separation involved a 40-min gradient elution using 98% A, 2% B to 68% A, and 32% B at 300 nL/min (A and B refer to mobile phases comprising 0.1% aqueous formic acid and 0.1% formic acid in acetonitrile, respectively). The MS spectrum (250 ms), followed by 10 MS/MS spectra (100-ms each) were acquired in data-dependent mode. Auto-calibration using 50 fmol of bovine serum albumin tryptic digests (KYA Technology, Tokyo, Japan) was performed for every four to five samples.

Raw data generated using Analysis TF 1.6 software (Sciex) were converted to mascot generic files by MS Data Converter (Sciex) and searched against an in-house mouse reference protein sequence database (Swiss-Prot; downloaded on July 7, 2020) using a Mascot search engine (v.2.6; Matrix Science, Boston, MA, United States). Peptide and MS/MS tolerances were set at ± 20 ppm and ± 0.1 Da, respectively. A maximum of two missed cleavages was allowed. Search settings involved cysteine carbamidomethylation as a fixed modification and the following variable modifications: deamidation of asparagine and/or glutamine, N-terminal glutamine to pyroglutamate, oxidation of methionine, oxidation of proline, and oxidation of lysine. The target false discovery rate (FDR) was set to < 1%. Proteins with more than three spectral matches were considered as identified proteins.

### Bioinformatics Analysis

Matrisome proteins were selected from all identified proteins using the Matrisome Annotator (http://matrisomeproject.mit.edu/analytical-tools/matrisome-annotator/) (Naba et al., 2016). The occupancy of each matrisome protein in controls was calculated based on the number of spectral counts (Lundgren et al., 2010), and the relative abundance of each protein was determined using the normalized spectral abundance factor (NSAF) (Zybailov et al., 2006). Statistical significance between the control and experimental groups was calculated using the Benjamini–Hochberg procedure (Benjamini and Hochberg, 1995) with log (fold change) > |0.2| and log (false discovery rate) > 2 as the threshold. The statistically significant proteins and proteins exclusively detected either in control or at day 14 were considered differentially expressed proteins (DEPs). Pathway- and process-enrichment analyses were performed using Metascape (Zhou et al., 2019) based on the following ontology sources: KEGG Pathway, GO Biological Processes, GO Cellular Components, GO Molecular Functions, Reactome Gene Sets, CORUM (comprehensive resource of mammalian protein complexes) WikiPathways, and Protein Analysis Through Evolutionary Relationships (PANTHER) Pathway. Terms with a *p*-value < 0.01, a minimum count of three, and an enrichment factor > 1.5 were grouped into clusters based on their similarities, with the most statistically significant term representing the cluster. PPI enrichment analysis was performed using STRING v11.5 (Szklarczyk et al., 2019). Local STRING network clusters were identified by hierarchical clustering of the full STRING network using an average linkage algorithm.

### Immunohistochemistry

Histological sections were deparaffinized in xylene and rehydrated with ethanol. Endogenous peroxidase activity was quenched with 3% hydrogen peroxide, blocked with 5% sheep or goat serum, and incubated with the primary antibody at 4°C overnight. Samples were washed three times with PBS-T and treated with an avidin-biotin complex kit (Vector Laboratories, Burlingame, CA, United States) according to manufacturer instructions, followed by visualization by incubation with 3,3′-diaminobenzidine tetrahydrochloride (DAB) (Vector Laboratories) and counter-staining with hematoxylin. The following primary antibodies were used: rabbit anti-collagen type II (1:100; 15943-1-AP), rabbit anti-collagen type III (1:200; 22734-1-AP), and rabbit anti-collagen type VI (1:100; 17023-1-AP) from ProteinTech (Manchester, United Kingdom); and rabbit anti-collagen type XII alpha 1 chain (1:100; HPA009143) from Merck (Darmstadt, Germany). Normal rabbit serum was used as a negative control. Histological sections were imaged using a BX53 microscope (Olympus, Tokyo, Japan) and processed using Fiji/ImageJ (NIH, Bethesda, MD, United States) and cellSens (Olympus) software.

### Picrosirius Red Staining

Picrosirius Red staining was performed, as previously described (Kaku et al., 2016). Histological sections were deparaffinized in xylene, rehydrated, and incubated with Picrosirius Red staining solution [1% (w/v) Sirius Red S in saturated aqueous picric acid] for 30 min, washed thoroughly with deionized water, dehydrated, and analyzed with a BX53 light microscope (Olympus) equipped with polarized filters.

### Statistical Analysis

Results are expressed as the means ± SD from three or more independent experiments. Statistical analysis for differences between the groups was performed using a two-tailed unpaired *t*-test with Welch’s correction using Prism 9 (GraphPad Software, San Diego, CA, United States) and a *p* value < 0.05 was considered significant.

## Results

### Mouse Molar Orthodontic Tooth Movement

**FIGURE 1:**
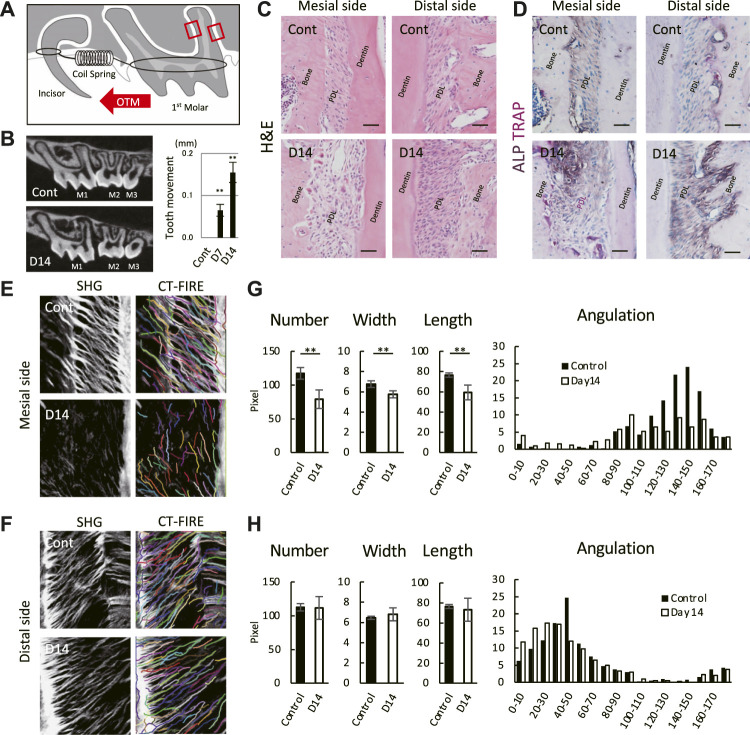
Mouse molar orthodontic tooth movement (OTM) and macroscale characteristics of collagenous fibers in the PDL. **(A)** Schematic of the OTM. Region of interests are indicated by red rectangles. **(B)** Representative micro-CT images of controls (Cont) and at day 14 (D14) of the OTM. The distance between the proximal surfaces of the 1^st^ and 2^nd^ molar. **p < 0.01 (*n* = 8, mean ± SD) **(C,D)** Staining at the mesial and distal sides of the PDL by hematoxylin and eosin **(H,E)** (*n* = 3) **(C)** and TRAP/ALP (*n* = 3) **(D)**. Scale bar: 50 μm **(E,F)** SHG microscopy image and collagenous fibers at the mesial **(E)** and distal **(F)** sides of the PDL. Individual fibers were detected using CT-FIRE. Quantification of detected fibers at the mesial **(G)** and distal **(H)** sides of the PDL. **p < 0.01 (*n* = 3, mean ± SD).

To apply static mechanical loading to the PDL, we placed a coil spring between the upper left 1^st^ molar and the upper incisors (Figure 1A). The mesial and distal sides of the PDL of the 1^st^ molar distal root were selected as the regions of interest. Micro-CT images and H&E staining showed the widening of the PDL space of the 1^st^ molar on day 14 (Figures 1B,C). An increased distance between the proximal surfaces of the 1^st^ and 2^nd^ molar confirmed mesial drift of the 1^st^ molar on day 14 (Figure 1B). Additionally, the number of TRAP-positive cells increased on the mesial side, whereas the ALP activity increased on the distal side on day 14 (Figure 1D), with high TRAP and ALP activities in the PDL, indicating persistent tooth movement.

### Macroscale Characteristics of Collagenous Fibers in Mouse Molar Periodontal Ligament

ECM organization at the macroscale showed individual collagenous fibers detected using SHG imaging and an automated tracking algorithm by CT-FIRE (Figures 1E,F). Controls showed well-aligned fibers on the mesial and distal sides of the PDL; however, the SHG signal for the mesial side of the PDL was difficult to detect on day 14 (Figure 1E bottom), and the fiber number, width, and length were significantly decreased (Figure 1G). The tendency for fiber angulation with respect to the cementum surface on the controls was not observed at day 14 on the mesial side. In contrast, the fiber number, width, length, and angulation remained unchanged at day 14 on the distal side (Figure 1H). These results indicate that the OTM used in this study induced destructive changes in macroscale fiber organization on the mesial side, whereas these changes were within the homeostatic balance on the distal side of the PDL.

### Extracellular Matrix-Oriented Proteome Analysis of Mouse Molar Periodontal Ligament

**FIGURE 2:**
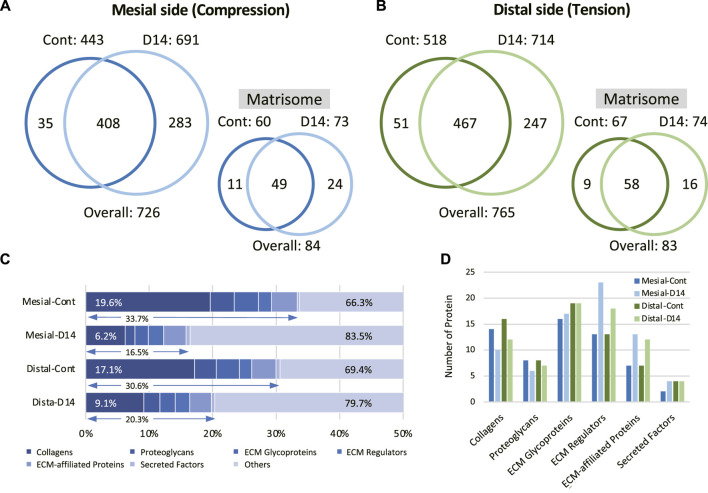
Identification and quantification of the mouse molar PDL matrisome proteins. Venn diagram of entire and matrisome proteins detected in control (Cont) and at day 14 (D14) of OTM on the mesial **(A)** and distal **(B)** sides of the PDL (*n* = 3) **(C)** Matrisome profile of the mouse molar PDL in each condition. Proportions of matrisome and collagens in reference to the entire proteome are shown. **(D)** Number of identified proteins in each matrisome subclass for each condition.

**FIGURE 3:**
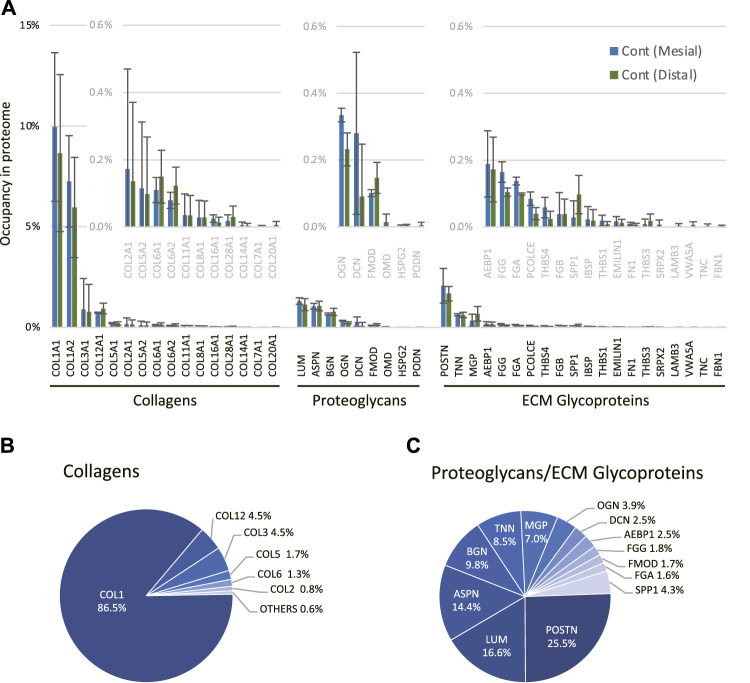
Profile of core matrisome proteins in the mouse molar PDL. **(A)** Profile of core matrisome (collagens, proteoglycans, and ECM glycoproteins) in the mouse molar PDL in controls. Insets are magnified graphs of the corresponding regions (*n* = 3, mean ± SD) **(B)** Profile of collagens in the mouse molar PDL in controls. **(C)** Profile of proteoglycans and ECM glycoproteins in the mouse molar PDL in controls.

Next, we performed a comprehensive view of the ECM proteome in the mouse molar PDL. The mesial and distal sides of the PDL were precisely dissected from the surrounding tissue to obtain PDL tissue-specific proteome (Supplementary Figure S1). On the mesial side, the number of identified proteins increased from 443 to 691 on day 14 (Figure 2A). Among the 726 identified proteins on the mesial side, 84 were matrisome proteins. On the distal side, the number of identified proteins increased from 518 to 714 on day 14 (Figure 2B), 83 of which were matrisome proteins. Additional unique proteins were detected at day 14 compared to the control on both mesial and distal sides. In controls, matrisome proteins accounted for ∼30% of the total spectral count (Figure 2C). Among matrisome proteins, collagens were the major sub-class, occupying ∼20% of the total spectral counts. The proportion of matrisome proteins decreased on day 14 at mesial and distal sides. The number of detected ECM regulators and ECM-affiliated proteins was increased, whereas that of collagens and proteoglycans was decreased on day 14 (Figure 2D). A complete list of core matrisome proteins in the controls comprised 13 collagen types with 16 chains, 9 proteoglycans, and 20 ECM glycoproteins (Figure 3A). Type I collagen (COL1A1 and COL1A2) was the most abundant matrisome protein in the PDL. accounting for 86.5% of the spectral count among collagens (Figure 3B). Type III and XII collagen (COL3A1 and COL12A1) each accounted for 4.5% of collagens, representing the 2^nd^ and 3^rd^ most abundant collagens in mouse molar PDL. Unexpectedly, type II collagen (COL2A1) was detected to account for 0.8% of collagens. Additionally, type V, VI, VII, VIII, XI, XIV, XVI, XX, and XXVIII collagens were identified (Figure 3A). Our proteome data revealed that lumican (LUM), asporin (ASPN), biglycan (BGN), and matrix gla protein (MGP) were the major proteoglycans, and periostin (POSTN) and tenascin-N (TNN) were the dominant ECM glycoproteins in the mouse molar PDL (Figure 3C).

### Changes in the Matrisome Profile of Mouse Molar Periodontal Ligament After Orthodontic Tooth Movement

**FIGURE 4:**
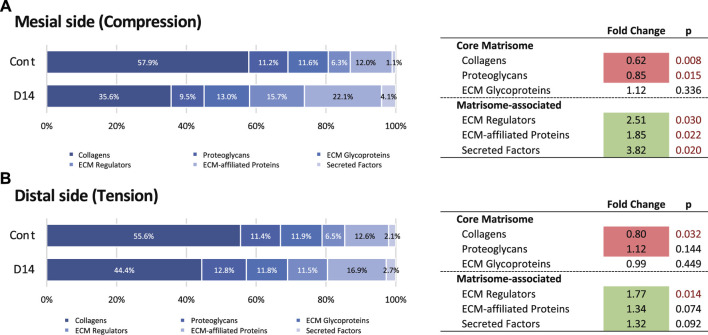
Changes in the matrisome profile at the mesial and distal sides of the PDL. Proportions of matrisome proteins and fold change of matrisome classes during OTM at mesial **(A)** and distal **(B)** sides of the PDL. Matrisome classes that significantly increased and decreased in proportion on day 14 (D14) compared with the control (Cont) are highlighted in green and red, respectively.

We then analyzed changes in the matrisome profile under mechanical loading at the mesial and distal sides. The proportion of collagens and proteoglycans in the mesial side of the PDL decreased on day 14, whereas that of matrisome-associated proteins increased (Figure 4A). A complete list of matrisome proteins on the mesial side showed that, on day 14, many ECM glycoproteins and ECM-associated proteins were detected that were not detected in controls (Supplementary Figure S2). The matrisome profile of the distal side of the PDL showed that the proportion of collagens decreased, while that of ECM regulators increased on day 14 (Figure 4B). A complete list of matrisome proteins on the distal side also showed that many ECM glycoproteins and ECM-associated proteins were detected on day 14 but were not found in controls (Supplementary Figure S3). We observed minor changes in the matrisome profile on the distal side compared with that of the mesial side of the PDL, which is consistent with the macroscale fiber characterization (Figures 1E–H).

### Identification of Matrisome-Oriented Differentially Expressed Proteins in Periodontal Ligament

**FIGURE 5:**
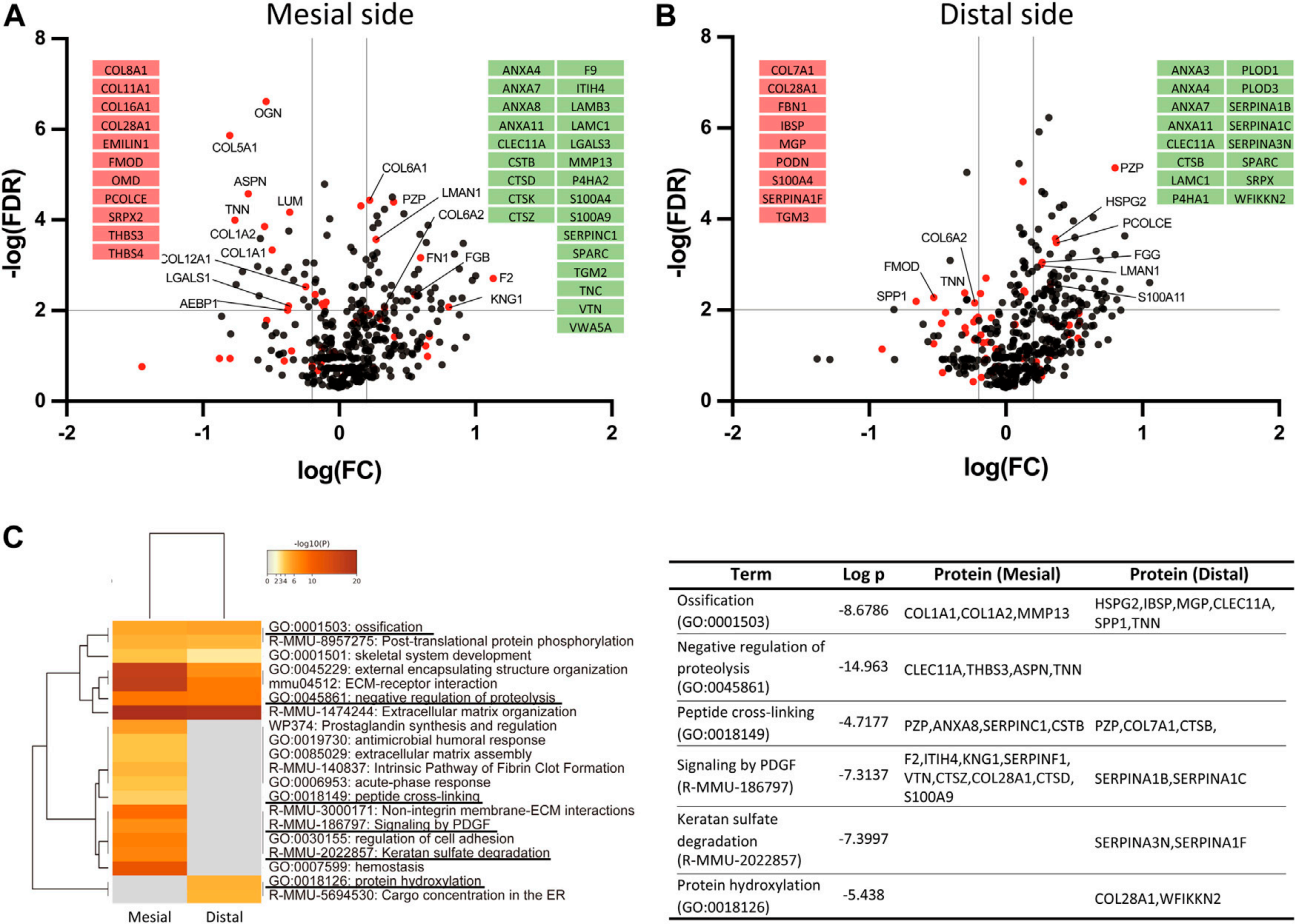
Identification of ECM-oriented differentially expressed proteins (DEPs) and enrichment analysis. Volcano plot of the DEPs at the mesial **(A)** and distal **(B)** sides of the PDL. Matrisome proteins are shown in red dots and other proteins are shown in black dots. The matrisome proteins exclusively detected only in control (red) or at day 14 (green) are shown in insets. **(C)** Pathway- and process-enrichment analyses of ECM-oriented DEPs in the mesial and distal sides of the PDL.

We next analyzed changes in individual matrisome proteins on day 14 and presented these using volcano plots (Figures 5A,B). On the mesial side, 53 DEPs (32 increased and 21 decreased in proportion) were identified (Figure 4A). The proportion of type VI collagen (COL6A1 and COL6A2) increased, but that of other collagens tended to decrease. The increased proportion of proteases (CTSK, CTSZ, and CTSD) and a matrix metalloproteinase (MMP13) indicated matrix degradation and bone resorption at the mesial side of the PDL. Proteins with increased proportion also included S100 proteins (A100A4, S100A9) and annexins (ANXA4, ANXA7, ANXA8, ANXA11). Proteins with decreased proportion include proteoglycans (ASPN, LUM, OGN, FMOD, OMD) and an ECM glycoprotein (TNN).

On the distal side, 35 DEPs (22 increased and 13 decreased in proportion) were identified (Figure 4B). The proportion of proteins associated with mineralization, such as matrix gla protein (MGP), osteopontin (SPP1), and bone sialoprotein (IBSP) was decreased. Notably, the proportion of collagen-modifying enzymes, including prolyl 4-hydroxylase (P4HA1) and lysyl hydroxylases (PLOD1 and PLOD3) was increased at the distal side of the PDL.

Among matrisome-oriented DEPs in the PDL, the expression of 9 proteins (ANXA11, ANXA4, ANXA7, CLEC11A, FGG, LAMC1, LMAN1, PZP, and SPARC) was increased and that of 2 proteins (FMOD and TNN) was decreased at both mesial and distal sides of the PDL.

### Enrichment Analysis Using Matrisome-Oriented Differentially Expressed Proteins

To better understand the molecular network regulating mechanical loading-induced ECM alterations in the PDL, we performed a matrisome-oriented protein-enrichment analysis (Figure 5C). Pathway-enrichment and process-enrichment analyses revealed that ossification (GO:0001503) and negative regulation of proteolysis (GO:0045861) were enriched at both mesial and distal sides of the PDL. PDGF signaling (R-MMU-186797) and keratan sulfate degradation (R-MMU-2022857) were enriched at the mesial side, and protein hydroxylation (GO:0018126) was enriched at the distal side.

We further performed protein-protein interaction (PPI) enrichment analysis of matrisome-oriented DEPs using the STRING database (Supplementary Figure S4). The selected PPI networks contributing to “ECM organization (R-MMU-1474244)” at the mesial side of the PDL showed that type I collagen (COL1A1 and COL1A2) was present at the center of the network and in close association with type VI collagen (COL6A1 and COL6A2) (Supplementary Figure S4A). Fibronectin1 (FN1) and SPARC were present at the interface of the “ECM organization” and “ECM-receptor interaction (R-MMU04512)” networks, likely playing a role as hub proteins. The selected PPI networks on the distal side of the PDL showed no strong relations at the network interface (Supplementary Figure S4B).

### Enrichment Analysis Using Differentially Expressed Proteins of Entire Proteome

Enrichment analysis was also performed for all identified proteins, using 431 DEPs of 726 proteins on the mesial side and 407 DEPs of 765 proteins on the distal side as inputs. The analytical method applied was identical to that used in the matrisome-oriented enrichment analysis. Although ECM-associated gene ontology terms were enriched at both the mesial and distal sides of the PDL, most enriched terms were associated with general cell metabolic processes (Supplementary Figure S5). PPI enrichment analysis using all DEPs showed no indicative results, although a matrisome protein-enriched cluster was clearly detected at the mesial and distal sides of the PDL (Supplementary Figures S6, S7).

### Collagen Distribution in the Mouse Molar Periodontal Ligament

**FIGURE 6:**
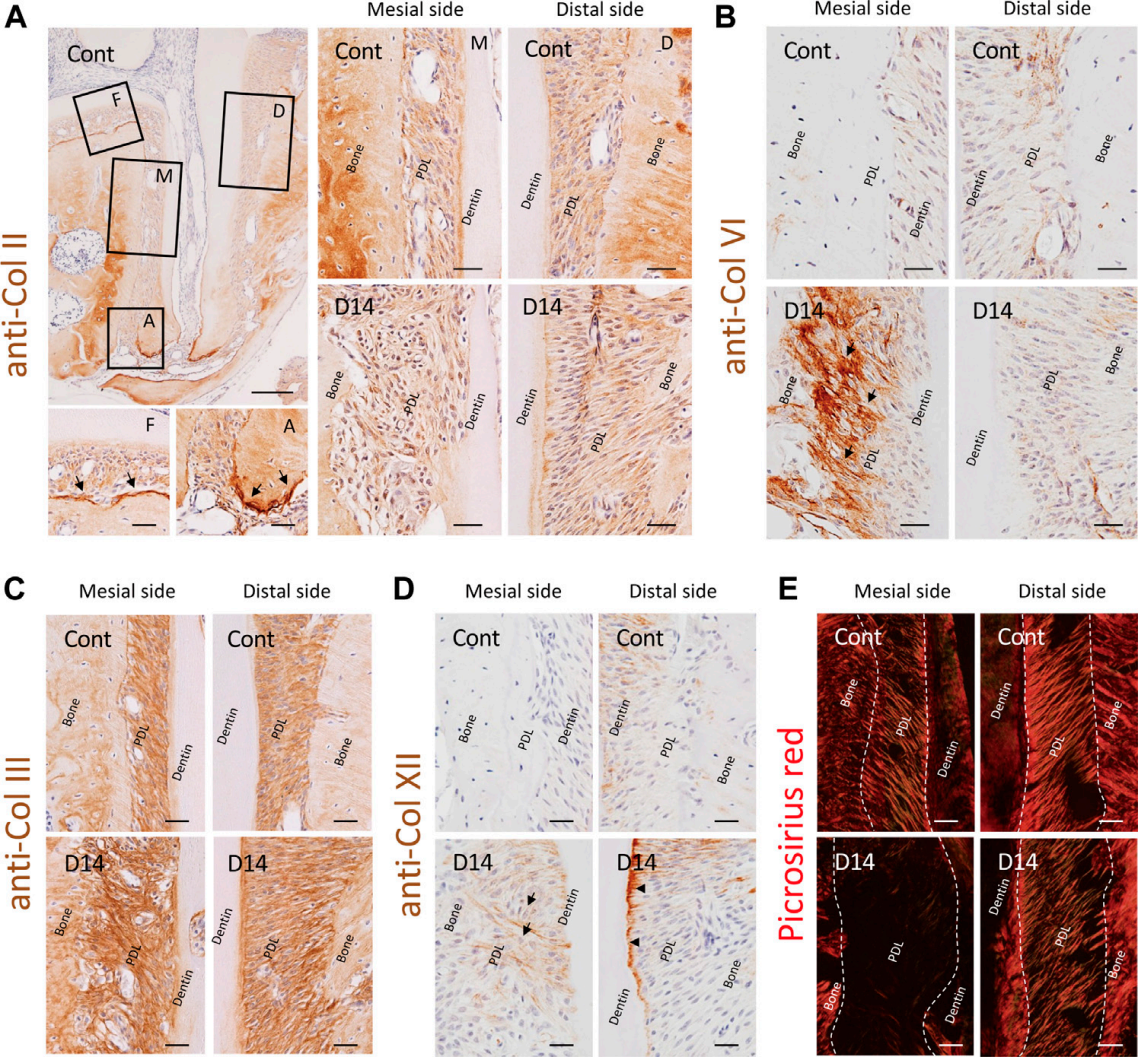
Effect of mechanical loading on collagen distribution in the mouse molar PDL. **(A)** Distribution of type II collagen in the PDL. Type II collagen was distributed throughout the PDL space, and intense staining was observed at the mineralized tissue surface, particularly at the bone surface of the furcation area and cementum surface of the apical area (arrows). F, furcation area; A, apical area; M, mesial side; D, distal side. **(B)** Distribution of type VI collagen in the PDL. Staining intensity of type VI collagen markedly increased along with that of the collagen fibers at the mesial side of the PDL, specifically at the bone side (arrows). **(C)** Distribution of type III collagen in the PDL. **(D)** Distribution of type XII collagen in the PDL. Staining intensity of type XII collagen was increased at the newly formed fibers on the mesial side of the PDL (arrows) and at the cementum surface on the distal side of the PDL (arrowheads) on day 14. **(E)** Polarized observation of picrosirius red staining, detecting matured collagen fibers. Scale bars: 200 μm at low magnification (a, top left) and 50 μm at high magnification (all other images).

Our proteomics data revealed a considerable amount of type II collagen in the PDL, which has not attracted attention so far. Our immunohistochemistry (IHC) data clearly showed that type II collagen was distributed throughout the PDL with intense signals at the mineralized-tissue surface, particularly at the bone surface of the furcation area and cementum surface of the apical area (Figure 6A). There were no notable changes in the staining intensity or distribution pattern of type II collagen on day 14, which is consistent with the results of the proteomic analysis (Supplementary Figures S2, S3).

In contrast, the staining intensity of type VI collagen was markedly increased at the mesial side of the PDL on day 14, whereas there was no noticeable change on the distal side of the PDL (Figure 6B). This correlated with the observed increase in abundance of type VI collagen at the mesial side of the PDL in response to mechanical loading according to proteomic analysis results (Figure 4A).

We also analyzed the 2^nd^ and 3^rd^ most abundant collagens, type III and XII collagens, using IHC, and analyzed type I collagen with picrosirius red staining. Type III collagen was distributed ubiquitously in the PDL space along without a noticeable change in the staining intensity on day 14 (Figure 6C). Type XII collagen was more abundant on the distal side of the PDL than on the mesial side of the PDL in controls (Figure 3A, Figure 6D), whereas the staining intensity increased on the newly formed fibers at the mesial side of the PDL and on the cementum surface at the distal side of the PDL on day 14. Picrosirius red staining showed trace signals at the mesial side of the PDL on day 14, indicative of the absence of matured type I collagen (Fig. 7e), despite the abundance of type III, VI, and XII collagens detected using IHC.

## Discussion

ECM is a principal component of tissues involving various functions; however, the composition of ECM in tissues is still largely unknown. One reason for this is that proteomic analysis in structurally complex tissue, such as the PDL, has been technically challenging (Aebersold and Mann, 2016). In the present study, we performed LMD and LC-MS/MS to comprehensively analyze the PDL tissue-specific proteome. Furthermore, matrisome-oriented data curation enabled the acquisition of a global ECM profile in the mouse molar PDL, to the best of our knowledge, for the first time. The detailed ECM profile of the PDL observed in this study provides fundamental insights that would be useful in understanding the role of the PDL tissue in health and diseases. Additionally, if the ECM composition of the PDL can be mimicked, it is possible to regulate cell behavior to enable tissue regeneration *in vivo* (Hussey et al., 2018). In fact, the ECM of the cultured PDL-derived cells was reported to produce PDL stem cell-like cells from iPS cells (Hamano et al., 2018). Therefore, the ECM profile of the PDL obtained in this study provides a foundation for successful biomimetic tissue regeneration.

Our proteome analysis revealed that the ECM of the PDL primarily comprises collagens; major collagen types are type I (86.5%), type III (4.5%), type XII (4.5%), and type V (1.7%) based on the spectral count (Figure 3B). Although we successfully illustrated the global ECM proteome of the PDL, there is a discrepancy in collagen proportion among previous reports, which indicate that type I, III, and V collagens occupied 75, 20, and 5% of the collagen in PDL, respectively (Butler et al., 1975; Yamauchi et al., 1986). This discrepancy is possibly due to the difference in the protein extraction method and identification strategies. The ECM proteins, especially collagens, are heavily cross-linked and have large molecular weight, which results in the insolubility during extraction (Byron et al., 2013). The earlier studies used acid extraction combined with chemical and enzymatic digestion and protein characterized by liquid chromatography (Butler et al., 1975; Yamauchi et al., 1986), while we employed the SDS extraction method with in-gel trypsin digestion for LC-MS/MS. Our method covered both core matrisome and matrisome-associated proteins, but the collagen profile of the tissue could be under-represented because of the incomplete extraction of heavily cross-linked proteins. Besides, matrisome analysis showed that the proportion of collagen decreased significantly (0.8-fold, p<0.05) at the distal side (Figure 4B), whereas characterization of the macroscale fiber did not indicate an alteration in this region (Figure 1H). One possible explanation is that the mechanical stress accelerates the collagen crosslinking in the PDL (Kaku et al., 2016) resulting in the decreased solubility of collagen (Taha and Naba, 2019). This may lead to the decreased proportion of collagen in the analytical sample from the distal side of PDL. To overcome technical limitations attributable to the insolubility of the ECM, several ECM-enrichment protocols including decellularization, deglycosylation, and chemical digestion have been developed (de Castro Bras et al., 2013; McCabe et al., 2021). Although these methods were shown to effectively enrich the core matrisome proteins, they also present a significant loss in the number of detected matrisome-associated proteins (Krasny and Huang, 2021). Therefore, an appropriate protein extraction protocol needs to be established on the basis of the biochemical nature of the target proteins. Further studies are needed to acquire a quantitatively accurate ECM profile of the PDL.

One of the key findings of this study is the presence of type II collagen in the PDL, which is in contrast to the results of previous studies citing this protein as a predominant collagen type in the cartilage (Ricard-Blum, 2011). Recent proteomic studies have identified type II collagens in mouse PDL (Connizzo et al., 2021) and rat PDL (Denes et al., 2020); however, these observations have not received attention. The increased type II collagen expression reported in cultured PDL-derived cells under cyclic tension force (Wescott et al., 2007) and hypoxic conditions (Li Q. et al., 2019) has been considered a result of their multipotent nature and chondrogenic differentiation potential (Seo et al., 2004). The distribution pattern of type II collagen, analyzed using IHC, indicated an association with bone/cementum apposition, similar to the fibrocartilage at enthesis, where type II collagen is present (Benjamin and Ralphs, 1998). Owing to the abundance of glycosylated hydroxylysine residues, type II collagen has larger molecular spacing than type I collagen (Grynpas et al., 1980), enabling it to hold more water and contributing to the dissipation of compressive forces (Bielajew et al., 2020). Collectively, type II collagen may play important roles in maintaining the tissue integrity of the PDL, and thus, further detailed investigation is required.

Our proteomic data showed that the proportion of type VI collagen increased at the mesial side of the PDL, but that of other collagens tended to decrease. PPI network analysis indicated a close association of type VI collagen with collagen fibrogenesis, particularly posttranslational modifications. Reportedly, type VI collagen interacts with the surrounding ECM and organizes the collagen fibril assembly and three-dimensional tissue architecture (Cescon et al., 2015). In periodontal tissue, type VI collagen localizes to the pericellular region surrounding fibroblasts and establishes extracellular microdomains that are associated with microfibrils and oxytalan fibers (Everts et al., 1998). Although details are still unclear, our results indicate that type VI collagen plays a crucial role in ECM alteration under mechanical loading in the PDL.

We noted the increased staining intensity of type XII collagen on the distal side of the PDL by IHC (Figure 6D); however, this was not detected in the proteome datasets (Supplementary Figure S3). The increased production of type XII collagen in response to the mechanical loading was reported in PDL tissue (Karimbux and Nishimura, 1995; Tsuzuki et al., 2016) and PDL-derived cells (Nemoto et al., 2010). In line with our observation, the fibril-associated type XII collagen has been shown to regulate collagen fibrillogenesis (Chiquet et al., 2014). Therefore, the increased staining intensity of type XII collagen at the cementum surface on the distal side of the PDL suggests enhanced fibril stabilization in this region. On the other hand, the discrepancy between our proteomic and IHC data on type XII collagen may be explained by the destruction of the tissue boundary during LMD, because the changes in the production of type XII collagen at the distal side of the PDL specifically occurred at the PDL-cementum boundary. Although the tissue-level proteome is a powerful tool to illustrate the tissue-specific ECM proteome, attention is required when the region-specific proteomic changes are of particular interest.

Our proteome analysis identified 2 calcium-binding protein families, S100 proteins and annexins; these proteins interact with each other and form complexes that exhibit biological activities (Miwa et al., 2008). S100A4 is highly expressed in PDL (Duarte et al., 1998) and is responsive to mechanical stress (Duarte et al., 1999). Furthermore, S100A4 downregulates osteogenic gene expression and upregulates matrix degradation-related gene expression in PDL cells (Zhou et al., 2015) as well as enhances osteoclast formation (Mah et al., 2015). We found that the proportion of S100A4 was increased on the mesial side, where the osteoclast recruitment was evident. In addition, annexin a2, an S100A4-binding partner, was also detected in PDL cells in high abundance (Xiong et al., 2016). Further, many S100 proteins (A100A4, S100A9) and annexins (ANXA4, ANXA7, ANXA8, ANXA11) were increased on the mesial side, and three annexins (ANXA4, ANXA7, ANXA11) were increased at both mesial and distal sides of the PDL. Although further investigations are needed, the S100-annexin complex may have reciprocal roles in PDL tissue maintenance under mechanical loading.

We observed increased production of collagen-modifying enzymes in response to mechanical loading, including that of prolyl hydroxylases (P4HA1/2), lysyl hydroxylases (PLOD1/3), and transglutaminases (TGM2/3). Type I collagen in the mouse molar PDL undergo distinct posttranslational modifications that significantly affect the formation of intermolecular cross-links and determine the structural and mechanical properties of the tissue (Hudson et al., 2017). We previously reported that the occlusal loading-induced mechanical stress enhances ECM maturation through the posttranslational modifications of collagen (Kaku et al., 2016). Our current data further support the notion that the posttranslational modifications of collagen play important roles in ECM organization in a mechanically challenging environment in the PDL.

This study reports putative mechanoresponsive proteins in PDL, whose roles regarding the ECM and/or PDL have not received attention. One example is the pregnancy zone protein (PZP), which is highly expressed in late-pregnancy serum and inhibits the activity of all four classes of proteinases (Wyatt et al., 2016). A recent study proposed that the PZP inhibits the aggregation of misfolded proteins that helps to maintain protein homeostasis during pregnancy (Cater et al., 2019). Our proteome data showed that the PZP increased 2.53-fold (*p* < 0.01) at the mesial side of the PDL and 6.22-fold (*p* < 0.01) at the distal side of the PDL. Other DEPs found on both sides of the PDL may also have undiscovered functions that involve mechano-transduction and ECM organization.

Although we identified numerous proteins via proteomic analysis, the number of detected proteins differed between the mesial and distal sides of the PDL in controls. Additionally, spectral counting values presented large standard deviations among biological replicates. These differences are likely because of the heterogeneity of the PDL tissue, given that the cell characteristics (Takimoto et al., 2015), fibrous network structure (Naveh et al., 2018), ECM composition (Denes et al., 2020; Connizzo et al., 2021), microvascular distribution (Sims et al., 1996), and response to mechanical stress (Takimoto et al., 2015; Kaku et al., 2016) differ between the respective regions of the PDL. The IHC data similarly showed that each protein had a distinct distribution pattern within the PDL. Collectively, it is necessary to consider the biomechanical and biochemical non-uniformity of the PDL (Connizzo et al., 2021). Indeed, non-uniformity of the PDL tissue is a critical factor that makes the clinical control and regeneration of this tissue extremely difficult.

In the ideal OTM theory, when the tooth is moved in the mesial direction, symmetric compression and tension forces are present at the mesial and distal sides of the PDL, respectively. However, the mesial movement of the tooth generates the compression force at the cervical region of the mesial and apical PDL, whereas the tension force is applied at the coronal two-thirds of the distal PDL (Li Z. et al., 2019). Additionally, the intrusive and/or tilting movement also occurs throughout the OTM process; therefore, the force is not completely uniform. This is one of the possible explanations for the overlapping of some of the DEPs, both at the mesial and distal sides of the PDL, while these may be involved simultaneously, maintaining PDL homeostasis in both compression and tension regions. As mentioned above, region-specific analysis of the ECM proteome and targeted analysis of specific proteins should be considered in future studies.

To enable the LMD of mineralized tissue-containing samples, we used PFA fixation for 24 h, followed by decalcification with 10% EDTA for 3 weeks at 4°C. Although fixation may affect the protein extraction efficiency, in theory, previous reports have shown that the number of identified proteins from formalin-fixed samples in the bottom-up proteomics is in concurrence with that obtained with fresh frozen tissue samples (Thompson et al., 2013). The decalcification may result in the loss of the mineral binding proteins (Chun et al., 2011; Salmon et al., 2017); however, this process is necessary for mineralized tissues, that is, enamel, dentin, cementum, and bone, and therefore, the effect on PDL is minimal. Special attention is required when interpreting the proteome data of the mineralized tissue using decalcified samples. Furthermore, we analyzed potential molecular networks involving ECM organization in the PDL using bioinformatics approaches. The use of ECM-oriented DEPs resulted in better resolution of the enriched terms, which included secretion, posttranslational modifications, and organization of the ECM; using the entire PDL proteome as an input resulted in enriched terms mainly related to general cellular metabolic processes. The process and pathway enrichment analysis showed that ossification (GO:0001503) and negative regulation of proteolysis (GO:0045861) were enriched at mesial and distal sides of the PDL. It has been well accepted that the cells in PDL maintain the adjacent alveolar bone by resorption and apposition of the bone matrix (Jiang et al., 2016; Moon et al., 2019; Lee et al., 2021). The enrichment of the “negative regulation of proteolysis” indicated that antagonizing mechanisms against ECM degradation were enhanced in response to the mechanical loading. We also showed that PDGF signaling (R-MMU-186797) involved ECM alteration on the mesial side of the PDL. PDGF is a widely studied growth factor (Giannobile, 1996) based on its abundance in platelet-rich plasma, demonstrating its capacity for periodontal-tissue regeneration (Fernandes and Yang, 2016). Moreover, PDGF stimulates proliferation, chemotaxis, and ECM production of human PDL-derived cells (Ojima et al., 2003) and acts synergistically with other growth factors to modulate the wound-healing process (Lynch et al., 1987). Our data suggest that the PDGF could be a potential target to achieve effective tissue regeneration of the PDL.

## Conclusion

Our ECM-oriented proteomics approach succeeded in illustrating the comprehensive ECM profile of the mouse molar PDL and its compositional changes following mechanical loading. Mechanical loading induced unique architectural changes in collagen fibers along with dynamic compositional changes in the matrisome profile, particularly involving ECM glycoproteins and matrisome-associated proteins. We identified several unique matrisome proteins that responded to different modes of mechanical loading in the PDL. Furthermore, the bioinformatics approach revealed potential molecular cues that maintain ECM homeostasis in a mechanically challenging environment. As a future prospect, the specific DEPs identified in this study need to be characterized extensively. Furthermore, the human PDL proteome needs to be clarified for the translation of the scientific findings to clinical practice. Our findings provide fundamental insights into the ECM network in the PDL, which are vital for precise clinical diagnosis and development of biomimetic tissue-regeneration strategies.

## Data Availability

The data that support the findings of this study are available within the article/Supplementary Material. The mass spectrometry proteomics data have been deposited to the jPOST (Japan ProteOme STandard Repository/Database, https://jpostdb.org) with the data set identifier JPST001418/PXD030430.
