# Supplementary material for: Extracellular Matrix-Oriented Proteomic Analysis of Periodontal Ligament Under Mechanical Stress
Source: Front Physiol. 2022 May 20;13:899699. doi: 10.3389/fphys.2022.899699 (PMC9163570; doi:10.3389/fphys.2022.899699)
Supplement: Supplementary file 1 [file DataSheet1.PDF]

Supplementary Material

1. Supplementary Figures and Tables

Supplementary Figure 1. Toluidine blue-stained cryosection after LMD

Supplementary Figure 2. Fold change of each matrisome protein at the compression side of PDL

Supplementary Figure 3. Fold change of each matrisome protein at the tension side of PDL

Supplementary Figure 4. PPI enrichment analysis of matrisome-oriented DEPs in PDL

Supplementary Figure 5. Enrichment analysis of entire DEPs in the PDL

Supplementary Figure 6. PPI Enrichment analysis of entire DEPs at the mesial side of the PDL

Supplementary Figure 7. PPI Enrichment analysis of entire DEPs at the distal side of the PDL

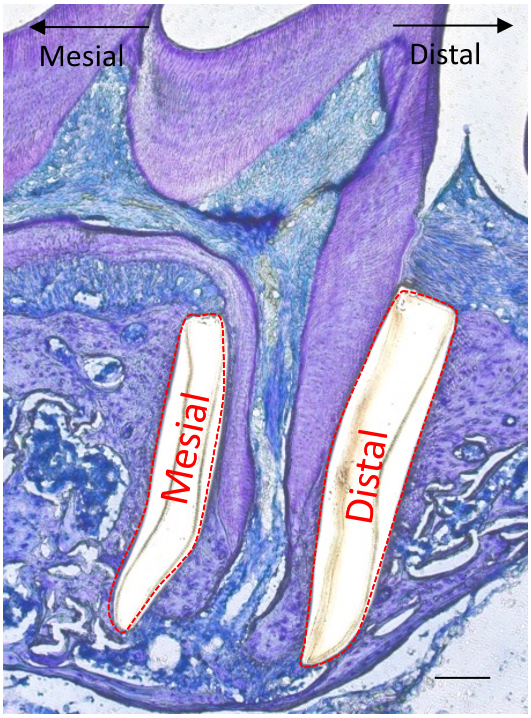

Fig. S1. Toluidine blue-stained cryosection after Laser microdissection (LMD).
Dissected regions - mesial and distal sides of the PDL - are indicated by dotted red lines.
Scale bar: 200 μm.

a

Core Matrisome

Collagens

Gene Symbol	Control (NSAF)	D14 (NSAF)	Fold Change	-log(FDR)
Col1a1	0.09951	0.03207	0.322	3.336
Col1a2	0.07253	0.02052	0.283	3.856
Col3a1	0.00889	0.00118	0.132	0.944
Col12a1	0.00731	0.00415	0.568	2.525
Col5a1	0.00214	0.00034	0.157	5.866
Col2a1	0.00172	0.00027	0.158	0.942
Col5a2	0.00114	0.00004	0.036	0.762
Col6a1	0.00110	0.00184	1.673	4.438
Col6a2	0.00079	0.00171	2.154	2.061
Col11a1	0.00036	0	0	
Col8a1	0.00029	0	0	
Col16a1	0.00025	0	0	
Col28a1	0.00018	0	0	
Col14a1	0.00005	0.00002	0.395	0.892

Proteoglycans

Gene Symbol	Control (NSAF)	D14 (NSAF)	Fold Change	-log(FDR)
Lum	0.01309	0.00567	0.433	4.170
Aspn	0.01052	0.00227	0.215	4.579
Bgn	0.00663	0.00496	0.749	2.145
Ogn	0.00335	0.00097	0.290	6.615
Dcn	0.00280	0.00125	0.447	1.110
Fmod	0.00101	0	0	
Omd	0.00014	0	0	
Hspg2	0.00002	0.00005	2.546	1.425

ECM Glycoproteins

Gene Symbol	Control (NSAF)	D14 (NSAF)	Fold Change	-log(FDR)
Postn	0.02065	0.00605	0.293	1.784
Tnn	0.00630	0.00108	0.171	3.999
Mgp	0.00348	0.00477	1.370	0.457
Aebp1	0.00189	0.00079	0.418	2.009
Fgg	0.00164	0.00237	1.438	4.310
Fga	0.00138	0.00183	1.329	1.763
Pcolce	0.00084	0	0	
Thbs4	0.00058	0	0	
Fgb	0.00038	0.00135	3.589	2.345
Spp1	0.00029	0.00124	4.312	1.227
Ibsp	0.00022	0.00016	0.738	0.858
Thbs1	0.00019	0.00032	1.703	1.940
Emilin1	0.00016	0	0	
Fn1	0.00010	0.00038	3.942	3.171
Thbs3	0.00009	0	0	
Srpx2	0.00008	0	0	
Vtn	0	0.00057	n/a	
Sparc	0	0.00014	n/a	
Tnc	0	0.00010	n/a	
Lamb3	0	0.00005	n/a	
Vwa5a	0	0.00003	n/a	
Lamc1	0	0.00001	n/a	

b

Matrisome-associated

ECM Regulators

Gene Symbol	Control (NSAF)	D14 (NSAF)	Fold Change	-log(FDR)
Serpinh1	0.01002	0.01070	1.067	0.541
Serpinf1	0.00403	0.00268	0.664	2.351
Serpina1d	0.00201	0.00139	0.694	0.680
Serpina3k	0.00194	0.00145	0.747	1.176
Serpina1c	0.00082	0.00147	1.796	0.724
Serpina1b	0.00076	0.00066	0.876	0.960
Serpina1e	0.00035	0.00037	1.054	0.731
Pzp	0.00034	0.00086	2.510	4.395
F10	0.00026	0.00053	2.012	1.804
Kng1	0.00008	0.00053	6.366	2.080
Hrg	0.00007	0.00004	0.633	0.789
F2	0.00006	0.00079	13.509	2.711
Plg	0.00004	0.00020	4.449	0.994
Ctsk	0	0.00096	n/a	
Tgm2	0	0.00068	n/a	
Ctsz	0	0.00039	n/a	
Ctsd	0	0.00029	n/a	
Mmp13	0	0.00013	n/a	
Cstb	0	0.00008	n/a	
Serpinc1	0	0.00007	n/a	
Itih4	0	0.00002	n/a	
F9	0	0.00002	n/a	
P4ha2	0	0.00001	n/a	

ECM-affiliated Proteins

Gene Symbol	Control (NSAF)	D14 (NSAF)	Fold Change	-log(FDR)
Anxa5	0.01371	0.01060	0.773	2.089
Anxa2	0.01169	0.00934	0.799	2.185
Lgals1	0.00557	0.00236	0.424	2.102
Anxa1	0.00382	0.00590	1.544	1.987
Anxa6	0.00304	0.00272	0.895	1.087
Hpx	0.00211	0.00196	0.933	1.045
Lman1	0.00025	0.00047	1.863	3.570
Anxa4	0	0.00105	n/a	
Lgals3	0	0.00038	n/a	
Anxa7	0	0.00019	n/a	
Anxa8	0	0.00012	n/a	
Anxa11	0	0.00011	n/a	
Clec11a	0	0.00002	n/a	

Secreted Factors

Gene Symbol	Control (NSAF)	D14 (NSAF)	Fold Change	-log(FDR)
S100a6	0.00324	0.00341	1.052	0.420
S100a11	0.00037	0.00169	4.583	1.431
S100a4	0	0.00125	n/a	
S100a9	0	0.00027	n/a	

: Increased after OTM

: Decreased after OTM

Fig. S2. Fold change of each matrisome protein at the compression side of PDL. Proteins which significantly increased and decreased on day 14 (D14) compared with the control were highlighted in green and red, respectively. NSAF; normalized spectral abundance factor: FDR; false discovery rate: OTM; orthodontic tooth movement.

a

Core Matrisome

Collagens

Gene Symbol	Control (NSAF)	D14 (NSAF)	Fold Change	-log(FDR)
Col1a1	0.08657	0.04396	0.508	1.505
Col1a2	0.05953	0.02988	0.502	1.618
Col12a1	0.00940	0.00572	0.608	1.809
Col3a1	0.00781	0.00682	0.873	0.750
Col5a1	0.00194	0.00066	0.339	1.710
Col6a1	0.00150	0.00087	0.583	1.740
Col2a1	0.00136	0.00128	0.943	0.743
Col6a2	0.00123	0.00073	0.592	2.159
Col5a2	0.00098	0.00084	0.860	0.744
Col11a1	0.00034	0.00025	0.715	0.940
Col28a1	0.00029	0	0	
Col8a1	0.00028	0.00024	0.837	0.745
Col16a1	0.00013	0.00009	0.661	0.527
Col20a1	0.00006	0.00010	1.674	0.864
Col14a1	0.00004	0.00003	0.837	0.748
Col7a1	0.00002	0	0	

Proteoglycans

Gene Symbol	Control (NSAF)	D14 (NSAF)	Fold Change	-log(FDR)
Lum	0.01131	0.00835	0.738	1.295
Aspn	0.01064	0.00666	0.625	1.845
Bgn	0.00769	0.00603	0.785	1.827
Ogn	0.00232	0.00152	0.657	1.460
Fmod	0.00147	0.00043	0.296	2.279
Dcn	0.00091	0.00262	2.883	1.465
Podn	0.00005	0	0	
Hspg2	0.00004	0.00009	2.312	3.568

ECM Glycoproteins

Gene Symbol	Control (NSAF)	D14 (NSAF)	Fold Change	-log(FDR)
Postn	0.01674	0.01327	0.792	0.624
Mgp	0.00677	0	0	
Tnn	0.00618	0.00309	0.500	2.377
Aebp1	0.00172	0.00117	0.682	1.284
Fgg	0.00105	0.00192	1.841	3.048
Fga	0.00099	0.00132	1.337	4.824
Spp1	0.00097	0.00021	0.221	2.187
Pcolce	0.0004	0.00093	2.341	3.481
Fgb	0.00038	0.00085	2.228	1.734
Thbs4	0.00024	0.00003	0.124	1.143
Ibsp	0.00019	0	0	
Thbs3	0.00018	0.00006	0.344	0.629
Emilin1	0.00012	0.00007	0.588	1.346
Thbs1	0.00008	0.00015	1.790	0.698
Fn1	0.00006	0.00016	2.908	1.671
Vwa5a	0.00006	0.00007	1.330	0.935
Lamb3	0.00004	0.00002	0.576	0.427
Fbn1	0.00003	0	0	
Tnc	0.00003	0.00004	1.149	0.363
Sparc	0	0.00041	n/a	
Srpx	0	0.00007	n/a	
Lamc1	0	0.00004	n/a	

b

Matrisome-associated

ECM Regulators

Gene Symbol	Control (NSAF)	D14 (NSAF)	Fold Change	-log(FDR)
Serpinh1	0.01095	0.01280	1.169	1.682
Serpinf1	0.00355	0.00299	0.842	1.148
Serpina1d	0.00205	0.00061	0.297	1.266
Serpina3k	0.00193	0.00187	0.972	0.364
Serpina1e	0.00029	0.00020	0.697	0.898
Kng1	0.00013	0.00028	2.107	0.976
F2	0.00012	0.00041	3.426	1.927
F10	0.00012	0.00022	1.821	0.552
Serpina1f	0.00011	0	0	
Pzp	0.00009	0.00056	6.281	5.122
Hrg	0.00008	0.00015	1.847	0.660
Tgm3	0.00006	0	0	
Plg	0.00005	0.00019	3.448	1.719
Serpina1c	0	0.00160	n/a	
Serpina1b	0	0.00049	n/a	
Plod1	0	0.00025	n/a	
Serpina3n	0	0.00016	n/a	
Ctsb	0	0.00011	n/a	
P4ha1	0	0.00007	n/a	
Plod3	0	0.00004	n/a	

ECM-affiliated Proteins

Gene Symbol	Control (NSAF)	D14 (NSAF)	Fold Change	-log(FDR)
Anxa5	0.01328	0.01172	0.882	1.146
Anxa2	0.01161	0.00758	0.653	2.366
Anxa1	0.00552	0.00396	0.717	2.705
Lgals1	0.00362	0.00379	1.048	0.366
Anxa6	0.00244	0.00331	1.356	2.425
Hpx	0.00122	0.00168	1.376	2.393
Lman1	0.00033	0.00060	1.818	3.003
Anxa4	0	0.00061	n/a	
Anxa7	0	0.00016	n/a	
Anxa3	0	0.00013	n/a	
Clec11a	0	0.00010	n/a	
Anxa11	0	0.00008	n/a	

Secreted Factors

Gene Symbol	Control (NSAF)	D14 (NSAF)	Fold Change	-log(FDR)
S100a6	0.00360	0.00130	0.362	1.942
S100a4	0.00111	0	0	
S100a11	0.00105	0.00225	2.139	2.573
S100a9	0.00052	0.00174	3.371	1.382
Wfikkn2	0	0.00006	n/a	

: Increased after OTM
: Decreased after OTM

Fig. S3. Fold change of each matrisome protein at the tension side of PDL. Proteins which significantly increased and decreased on day 14 (D14) compared with the control were highlighted in green and red, respectively. NSAF; normalized spectral abundance factor: FDR; false discovery rate: OTM; orthodontic tooth movement.

Thant et al. Fig. S4

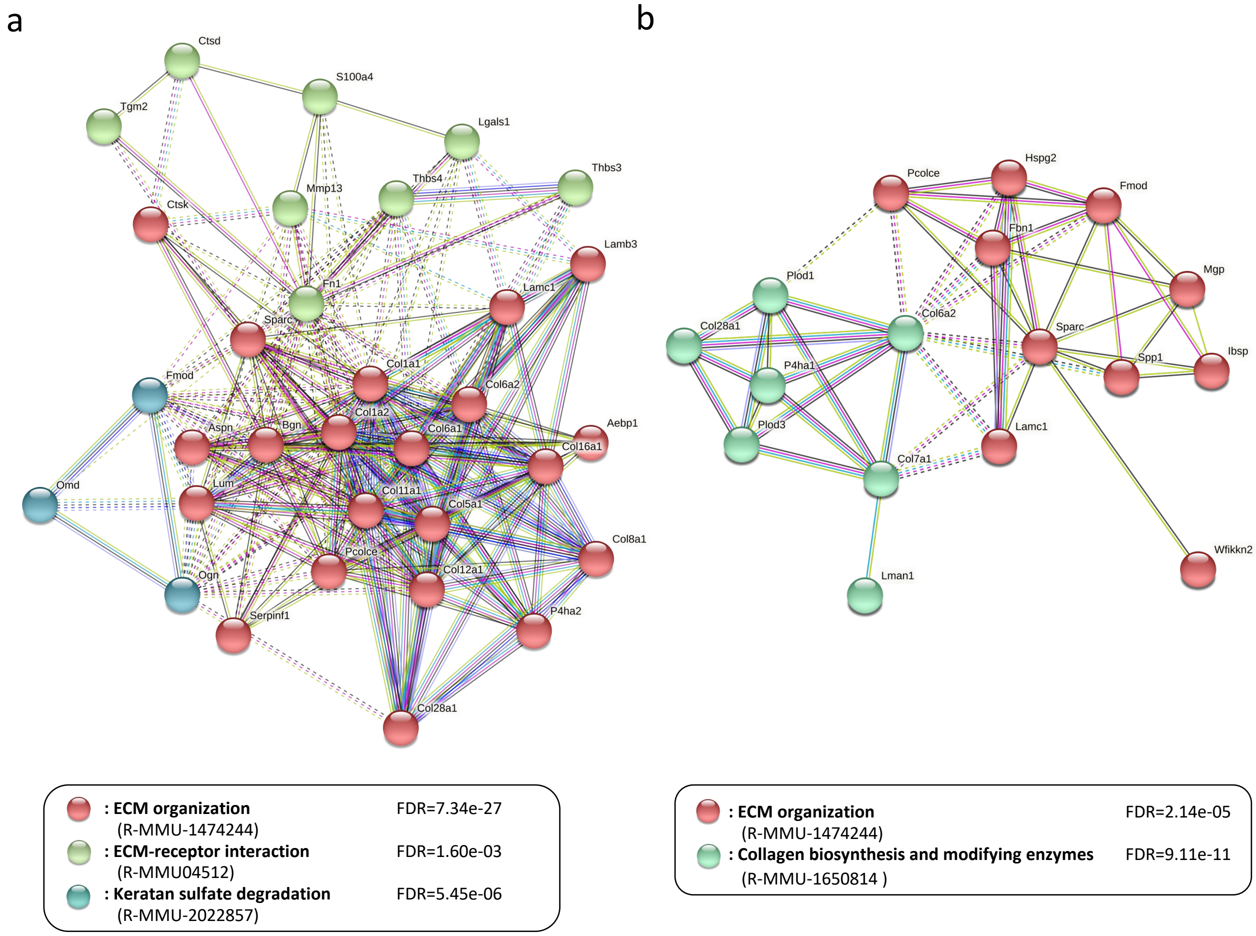

Fig. S4. Protein-protein interaction (PPI) enrichment analysis of matrisome-oriented DEPs in PDL. PPI network analysis of matrisome-oriented DEPs at the mesial side (a) and distal side (b) of the PDL. FDR; false discovery rate.

Thant et al. Fig. S5

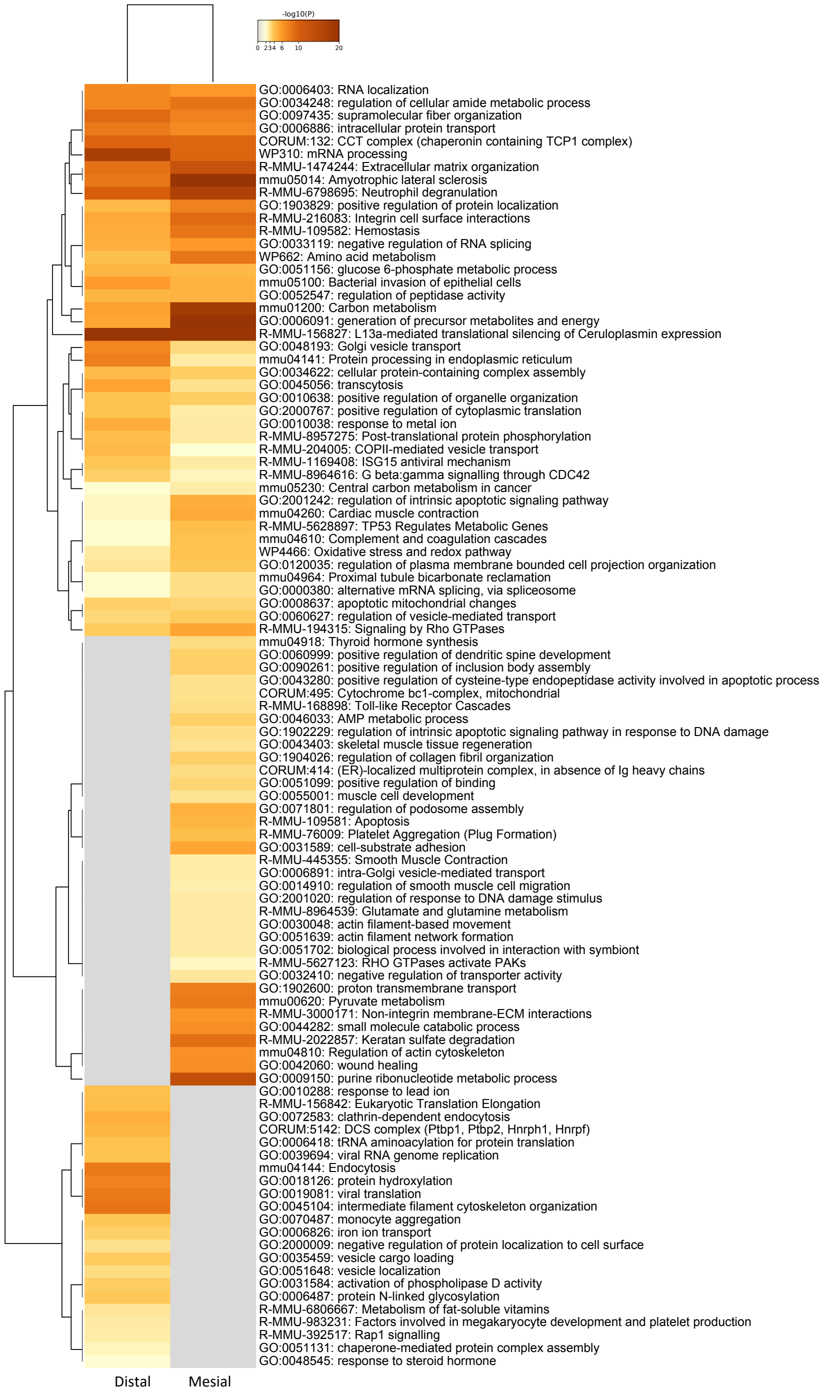

Fig. S5. Enrichment analysis of entire DEPs in the PDL. (a) Pathway- and process-enrichment analyses of entire DEPs in the mesial and distal sides of the PDL.

Thant et al. Fig. S6

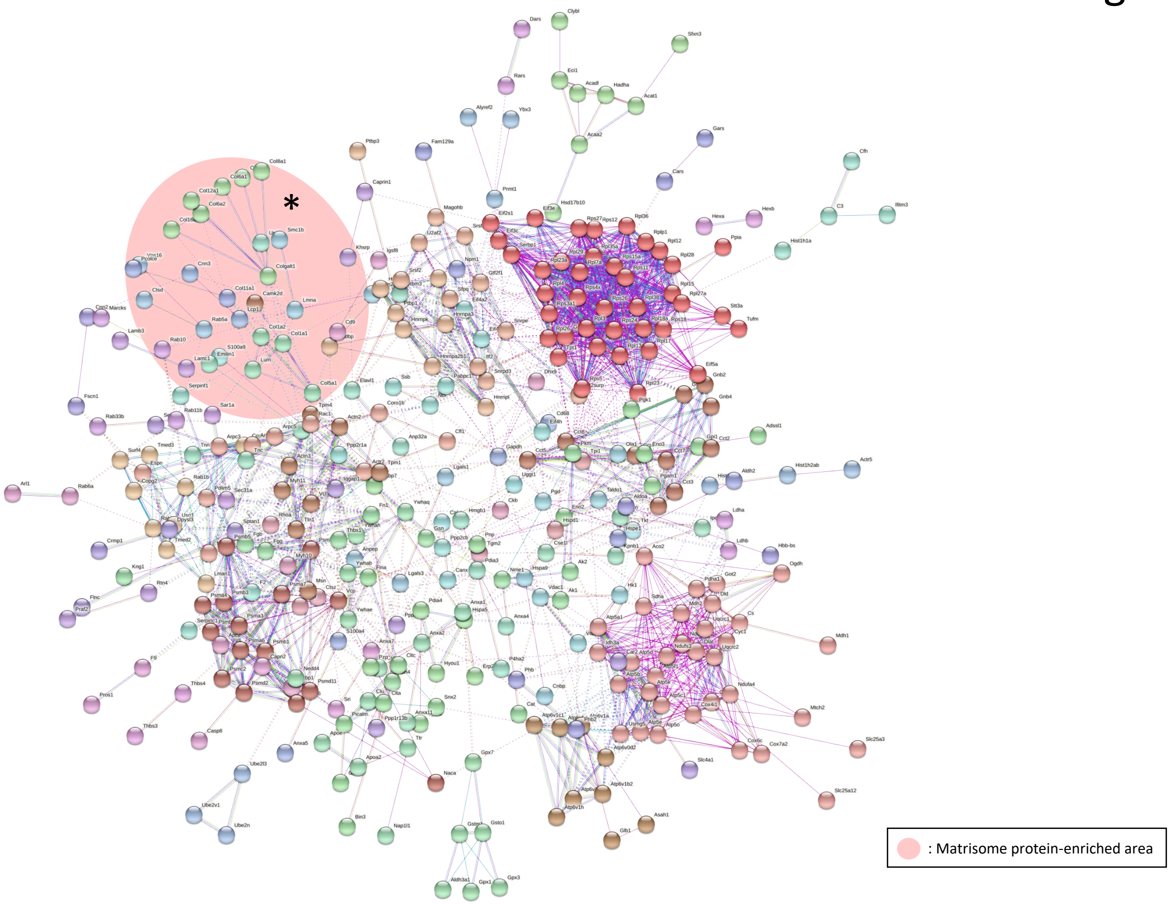

Local network cluster of DEPs at the mesial side PDL

#Term ID	Term description	FDR	Matching proteins in network
CL:5816	SRP-dependent cotranslational protein targeting to membrane	1.98E-18	Rps11,Rps5,Rplp1,Rps18,Gnb2l1,Rps26,Rps3a1,Rpl28,Rps4x,Rpl4,Rpl18a,Rpl26,Rpl17,Rpl15,Rpl36,Rps17,Rpl29,Rpl3,Rpl23,Rpl23a,Rpl7a,Rpl38,Rpl35a,Rpl13a,Rpl12,Rps15a,Rpl27a,Rps24,Rps27
CL:5809	SRP-dependent cotranslational protein targeting to membrane, and Ribosomal protein S30	2.16E-18	Rps11,Rps5,Rplp1,Rps18,Gnb2l1,Rps26,Rps3a1,Rpl28,Rps4x,Rpl4,Rpl18a,Rpl26,Rps12,Rpl17,Rpl15,Rpl36,Rps17,Rpl29,Rpl3,Rpl23,Rpl23a,Rpl7a,Rpl38,Tpt1,Rpl35a,Rpl13a,Rpl12,Rps15a,Rpl27a,Rps24,Rps27
CL:5810	cytosolic ribosome	3.39E-18	Rps11,Rps5,Rplp1,Rps18,Gnb2l1,Rps26,Rps3a1,Rpl28,Rps4x,Rpl4,Rpl18a,Rpl26,Rpl17,Rpl15,Rpl36,Rps17,Rpl29,Rpl3,Rpl23,Rpl23a,Rpl7a,Rpl38,Tpt1,Rpl35a,Rpl13a,Rpl12,Rps15a,Rpl27a,Rps24,Rps27
CL:5761	mixed, incl. cytosolic ribosome, and Ribosomal protein L7Ae/L30e/S12e/Gadd45 family	6.75E-15	Rps11,Rps5,Rplp1,Rps18,Gnb2l1,Rps26,Rps3a1,Rpl28,Rps4x,Rpl4,Rpl18a,Rpl26,Rps12,Rpl17,Rpl15,Rpl36,Rps17,Rpl29,Rpl3,Naca,Rpl23,Rpl23a,Rpl7a,Rpl38,Tpt1,Rpl35a,Rpl13a,Rpl12,Rps15a,Rpl27a,Rps24,Rps27
CL:5817	cytosolic large ribosomal subunit	2.76E-10	Rplp1,Rpl28,Rpl4,Rpl18a,Rpl17,Rpl15,Rpl36,Rpl29,Rpl3,Rpl23,Rpl23a,Rpl7a,Rpl38,Rpl35a,Rpl13a,Rpl12,Rpl27a
CL:23123	Carbon metabolism, and Pyruvate metabolism	4.10E-10	Ogdh,Eno2,Cs,Pgam1,Mdh2,Sdha,Tkt,Aco2,Taldo1,Ldhb,Pdha1,Got2,Dlat,Pkm,Gpi1,Eno3,Pgk1,Pgd,Aldoa,Mdh1,Ldha,Dld,Idh3a,Tpi1
CL:13973	Extracellular matrix organization, and CSPG repeat	4.10E-09	Serpinf1,Col6a1,Col6a2,Col1a1,Fkbp11,Lamb3,Vtn,P4ha2,Aspn,Thbs4,Lamc1,Col5a1,Itgav,Thbs3,Emilin1,Col1a2,Pcolce,Col16a1,Tnn,Thbs1,Colgalt1,Fn1,Col12a1,Col8a1,Col11a1,Tnc,Col28a1
CL:5821	cytosolic large ribosomal subunit	9.11E-09	Rplp1,Rpl28,Rpl4,Rpl18a,Rpl17,Rpl15,Rpl36,Rpl3,Rpl23,Rpl23a,Rpl7a,Rpl38,Rpl35a,Rpl13a,Rpl27a
CL:13974	Extracellular matrix organization	1.61E-07	Serpinf1,Col6a1,Col6a2,Col1a1,Fkbp11,Lamb3,Vtn,P4ha2,Lamc1,Col5a1,Itgav,Emilin1,Col1a2,Pcolce,Col16a1,Tnn,Thbs1,Colgalt1,Fn1,Col12a1,Col8a1,Col11a1,Tnc,Col28a1
CL:7322	Cross-presentation of soluble exogenous antigens (endosomes)	7.97E-07	Psmb4,Psmd2,Psmb1,Psmd11,Psmb6,Psma6,Psmc6,Psmb5,Psma7,Psmc2,Psma4,Psmb3,Psma3
CL:9505	Electron transport chain, and mitochondrial proton-transporting ATP synthase complex	8.00E-07	Cox5a,Ndufs3,Cox6c,Atp5e,Cyc1,Atp5o,Atp5b,Atp5a1,Uqcrc1,Ndufs1,Ndufa4,Uqcrc2,Cox4i1,Cox7a2,Atp5k,Usmg5,Atp5d,Atp5c1,Atp5f1
CL:8008	RNA recognition motif. (a.k.a. RRM, RBD, or RNP domain), and KH domain	2.18E-06	Ilf2,Khsrp,Caprin1,Sfpq,Dhx9,Hnrnpk,Hnrnpa1,Tardbp,Elavl1,Hnrnpc,Hnrnpa3,Hnrnpa2b1,Gapdh,Ptbp1,Hnrnpl,Purb
CL:7324	Proteasome	2.39E-06	Psmb4,Psmd2,Psmb1,Psmd11,Psmb6,Psma6,Psmc6,Psmb5,Psma7,Psmc2,Psma4,Psma3
CL:5824	cytosolic large ribosomal subunit	2.93E-06	Rpl28,Rpl4,Rpl18a,Rpl15,Rpl36,Rpl3,Rpl23,Rpl23a,Rpl38,Rpl35a,Rpl13a,Rpl27a
CL:14216	Collagen biosynthesis and modifying enzymes	5.17E-06	Serpinf1,Col6a1,Col6a2,Col1a1,Fkbp11,P4ha2,Col5a1,Col1a2,Pcolce,Col16a1,Colgalt1,Col8a1,Col11a1,Col28a1
CL:8006	RNA recognition motif. (a.k.a. RRM, RBD, or RNP domain), and KH domain	5.17E-06	Ilf2,Khsrp,Caprin1,Sfpq,Cnbp,Dhx9,Hnrnpk,Hnrnpa1,Tardbp,Elavl1,Hnrnpc,Hnrnpa3,Hnrnpa2b1,Gapdh,Ptbp1,Hnrnpl,Purb
CL:7130	proteasome complex, and Chaperone	1.60E-05	Cct3,Psmb4,Psmd2,Psmb1,Psmd11,Psmb6,Psma6,Psmc6,Psmb5,Cct5,Cct8,Psma7,Psmc2,Cct6a,Cct7,Psma4,Cct2,Psmb3,Psma3,Gtse1,Cct4
CL:5906	cytosolic small ribosomal subunit	7.84E-05	Rps11,Rps5,Rps18,Gnb2l1,Rps26,Rps3a1,Rps4x,Rpl26,Rps17,Rps15a,Rps24
CL:14217	Collagen biosynthesis and modifying enzymes	8.17E-05	Col6a1,Col6a2,Col1a1,P4ha2,Col5a1,Col1a2,Pcolce,Col16a1,Colgalt1,Col8a1,Col11a1,Col28a1
CL:7325	Proteasome	8.17E-05	Psmb4,Psmb1,Psmb6,Psma6,Psmc6,Psmb5,Psma7,Psmc2,Psma4,Psma3
CL:8013	mixed, incl. negative regulation of mRNA metabolic process, and paraspeckles	8.17E-05	Ilf2,Sfpq,Dhx9,Hnrnpk,Hnrnpa1,Elavl1,Hnrnpc,Hnrnpa3,Hnrnpa2b1,Gapdh,Ptbp1,Hnrnpl
CL:5826	cytosolic large ribosomal subunit	0.00011	Rpl28,Rpl4,Rpl18a,Rpl15,Rpl3,Rpl23,Rpl23a,Rpl38,Rpl13a,Rpl27a
CL:9671	mitochondrial proton-transporting ATP synthase complex	0.00017	Atp5e,Atp5o,Atp5b,Atp5a1,Atp5k,Usmg5,Atp5d,Atp5c1,Atp5f1
CL:7305	Regulation of ornithine decarboxylase (ODC), and proteasome binding	0.00041	Psmb4,Psmd2,Psmb1,Psmd11,Psmb6,Psma6,Psmc6,Psmb5,Psma7,Psmc2,Psma4,Psmb3,Psma3,Gtse1
CL:9674	proton-transporting ATP synthase activity, rotational mechanism	0.00042	Atp5e,Atp5o,Atp5b,Atp5a1,Atp5k,Atp5d,Atp5c1,Atp5f1
CL:23127	Pentose phosphate pathway, and pyruvate metabolic process	0.00043	Eno2,Pgam1,Tkt,Taldo1,Ldhb,Pkm,Gpi1,Eno3,Pgk1,Pgd,Aldoa,Ldha,Tpi1
CL:8016	mixed, incl. negative regulation of mRNA metabolic process, and paraspeckles	0.0012	Ilf2,Sfpq,Dhx9,Hnrnpk,Hnrnpc,Hnrnpa3,Hnrnpa2b1,Gapdh,Ptbp1,Hnrnpl
CL:5911	cytosolic small ribosomal subunit	0.0016	Rps11,Rps5,Rps18,Rps26,Rps3a1,Rpl26,Rps17,Rps15a,Rps24
CL:7240	Chaperonin TCP-1, conserved site	0.002	Cct3,Cct5,Cct8,Cct6a,Cct7,Cct2,Cct4
CL:5828	cytosolic large ribosomal subunit	0.0023	Rpl28,Rpl4,Rpl18a,Rpl15,Rpl3,Rpl23,Rpl23a,Rpl13a
CL:8017	mixed, incl. CRD-mediated mRNA stabilization, and RNPHF zinc finger	0.0033	Ilf2,Dhx9,Hnrnpk,Hnrnpc,Hnrnpa3,Hnrnpa2b1,Gapdh,Ptbp1,Hnrnpl
CL:5830	cytosolic large ribosomal subunit	0.0067	Rpl28,Rpl4,Rpl18a,Rpl15,Rpl3,Rpl23,Rpl13a
CL:23265	TCA cycle, and Aminotransferase	0.008	Ogdh,Cs,Mdh2,Sdha,Aco2,Pdha1,Got2,Dlat,Mdh1,Dld,Idh3a
CL:10048	proton-transporting V-type ATPase complex	0.0094	Atp6v1b2,Atp6v1e1,Atp6v1d,Atp6v1c1,Atp6v0d2,Atp6v1h,Atp6v1a
CL:10051	proton-transporting ATPase activity, rotational mechanism	0.0246	Atp6v1b2,Atp6v1e1,Atp6v1d,Atp6v1c1,Atp6v1h,Atp6v1a

Fig. S6. PPI enrichment analysis of the entire DEPs at the mesial side of the PDL. The results were generated by STRING v.11.5. *: Matrisome protein-enriched area

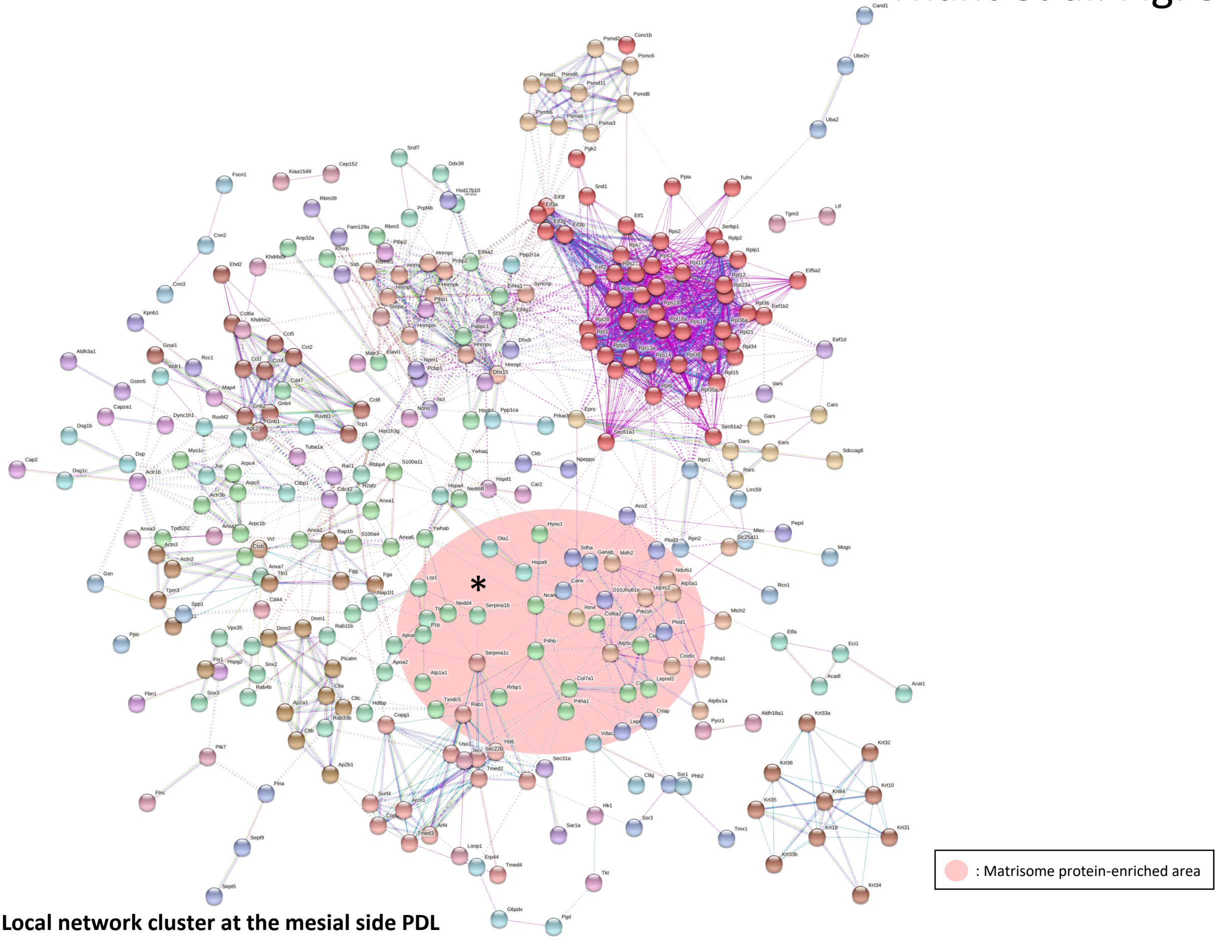

Local network cluster at the mesial side PDL

#Term ID	Term description	FDR	Matching proteins in network
CL:5815	cytosolic ribosome	4.00E-22	Rps11,Rplp1,Rps18,Rps14,Rps3a1,Rpl6,Rpl28,Rps3,Rpl21,Eef2,Rpl18a,Rps21,Rps7,Rpl17,Rpl15,Rpl36,Rpl3,Rplp2,Rplp0,Rpl34,Rps2,Rpl23a,Rpl11,Rps8,Rpl38,Rps19,Rpl36a,Rpl35a,Rpl13a,Rpl12,Rps13,Rps27
CL:5816	SRP-dependent cotranslational protein targeting to membrane	5.49E-22	Rps11,Rplp1,Rps18,Rps14,Rps3a1,Rpl6,Rpl28,Rps3,Rpl21,Eef2,Rpl18a,Rps21,Rps7,Rpl17,Rpl15,Rpl36,Rpl3,Rplp2,Rplp0,Rpl34,Rps2,Rpl23a,Rpl11,Rps8,Rpl38,Rps19,Rpl35a,Rpl13a,Rpl12,Rps13,Rps27
CL:5809	SRP-dependent cotranslational protein targeting to membrane, and Ribosomal protein S30	7.00E-22	Rps11,Rplp1,Rps18,Rps14,Rps3a1,Rpl6,Rpl28,Rps3,Rpl21,Eef2,Rpl18a,Rps21,Rps12,Rps7,Rpl17,Rpl15,Rpl36,Rpl3,Rplp2,Rplp0,Rpl34,Rps2,Rpl23a,Rpl11,Rps8,Rpl38,Rps19,Rpl36a,Rpl35a,Rpl13a,Rpl12,Rps13,Rps27
CL:5774	cytosolic ribosome, and Elongation factor	7.34E-20	Rps11,Rplp1,Rps18,Rps14,Rps3a1,Rpl6,Rpl28,Rps3,Rpl21,Eef2,Rpl18a,Rps21,Rps12,Rps7,Rpl17,Rpl15,Rpl36,Rpl3,Rplp2,Rplp0,Rpl34,Eef1d,Rps2,Rpl23a,Rpl11,Rps8,Rpl38,Rps19,Rpl36a,Rpl35a,Rpl13a,Eef1b2,Rpl12,Rps13,Rps27
CL:5817	cytosolic large ribosomal subunit	5.43E-12	Rplp1,Rpl6,Rpl28,Rpl21,Rpl18a,Rpl17,Rpl15,Rpl36,Rpl3,Rplp2,Rplp0,Rpl34,Rpl23a,Rpl11,Rpl38,Rpl35a,Rpl13a,Rpl12
CL:8013	mixed, incl. negative regulation of mRNA metabolic process, and paraspeckles	7.11E-12	Ptbp2,Nono,Dhx9,Hnrnpk,Hnrnpa1,Hnrnpu,Rbmxl1,Pcbp1,Syncrip,Srsf7,Pcbp2,Elavl1,Hnrnpc,Hnrnpm,Matr3,Ptbp1,Hnrnpf,Hnrnpl
CL:5821	cytosolic large ribosomal subunit	1.17E-11	Rplp1,Rpl6,Rpl28,Rpl21,Rpl18a,Rpl17,Rpl15,Rpl36,Rpl3,Rplp2,Rplp0,Rpl34,Rpl23a,Rpl11,Rpl38,Rpl35a,Rpl13a
CL:8008	RNA recognition motif. (a.k.a. RRM, RBD, or RNP domain), and KH domain	5.66E-10	Khsrp,Ptbp2,Nono,Dhx9,Hnrnpk,Hnrnpa1,Hnrnpu,Rbmxl1,Pcbp1,Syncrip,Srsf7,Pcbp2,Elavl1,Hnrnpc,Hnrnpm,Matr3,Ptbp1,Hnrnpf,Hnrnpl
CL:8016	mixed, incl. negative regulation of mRNA metabolic process, and paraspeckles	1.61E-09	Ptbp2,Nono,Dhx9,Hnrnpk,Hnrnpu,Pcbp1,Syncrip,Srsf7,Pcbp2,Hnrnpc,Hnrnpm,Matr3,Ptbp1,Hnrnpf,Hnrnpl
CL:8017	mixed, incl. CRD-mediated mRNA stabilization, and RNPHF zinc finger	6.32E-08	Dhx9,Hnrnpk,Hnrnpu,Pcbp1,Syncrip,Srsf7,Pcbp2,Hnrnpc,Hnrnpm,Matr3,Ptbp1,Hnrnpf,Hnrnpl
CL:5824	cytosolic large ribosomal subunit	8.51E-08	Rpl6,Rpl28,Rpl21,Rpl18a,Rpl15,Rpl36,Rpl3,Rpl34,Rpl23a,Rpl11,Rpl38,Rpl35a,Rpl13a
CL:8018	mixed, incl. pre-mRNA binding, and RNPHF zinc finger	1.72E-06	Hnrnpk,Hnrnpu,Pcbp1,Srsf7,Pcbp2,Hnrnpc,Hnrnpm,Matr3,Ptbp1,Hnrnpf,Hnrnpl
CL:5906	cytosolic small ribosomal subunit	3.28E-06	Rps11,Rps18,Rps14,Rps3a1,Rps3,Eef2,Rps21,Rps7,Rps2,Rps8,Rps19,Rps13
CL:5826	cytosolic large ribosomal subunit	4.57E-06	Rpl6,Rpl28,Rpl21,Rpl18a,Rpl15,Rpl3,Rpl34,Rpl23a,Rpl11,Rpl38,Rpl13a
CL:8020	mixed, incl. RNPHF zinc finger, and mRNA CDS binding	6.17E-05	Hnrnpk,Hnrnpu,Pcbp1,Pcbp2,Hnrnpc,Hnrnpm,Ptbp1,Hnrnpf,Hnrnpl
CL:5911	cytosolic small ribosomal subunit	9.91E-05	Rps11,Rps18,Rps14,Rps3a1,Rps3,Rps21,Rps7,Rps8,Rps19,Rps13
CL:7240	Chaperonin TCP-1, conserved site	0.0021	Cct5,Cct8,Cct6a,Cct7,Cct2,Tcp1,Cct4
CL:5828	cytosolic large ribosomal subunit	0.0022	Rpl6,Rpl28,Rpl18a,Rpl15,Rpl3,Rpl34,Rpl23a,Rpl13a
CL:14216	Collagen biosynthesis and modifying enzymes	0.0026	Col6a2,Plod3,P4ha1,Plod1,Leprel2,Pcolce,Colgalt1,Crtap,Lepre1,Col7a1,Col28a1
CL:7324	Proteasome	0.0029	Psmd2,Psmd11,Psmb6,Psma6,Psmd6,Psmc6,Psmd1,Psmd8,Psma3
CL:8022	mixed, incl. mRNA CDS binding, and RNPHF zinc finger	0.003	Hnrnpk,Hnrnpu,Hnrnpc,Hnrnpm,Ptbp1,Hnrnpf,Hnrnpl
CL:7130	proteasome complex, and Chaperone	0.0055	Psmd2,Psmd11,Psmb6,Hspa4,Psma6,Psmd6,Psmc6,Cct5,Cct8,Psmd1,Cct6a,Cct7,Cct2,Psmd8,Tcp1,Psma3,Cct4
CL:5830	cytosolic large ribosomal subunit	0.0063	Rpl6,Rpl28,Rpl18a,Rpl15,Rpl3,Rpl34,Rpl13a
CL:13974	Extracellular matrix organization	0.0082	Col6a2,Plod3,Cd44,P4ha1,Plod1,Leprel2,Lamc1,Fbn1,Pcolce,Tnn,Colgalt1,Crtap,Lepre1,Col7a1,Spp1,Col28a1,Hspg2
CL:18051	mixed, incl. S-100/ICaBP type calcium binding domain, and Annexin	0.0082	S100a4,Anxa11,Anxa1,S100a11,Anxa3,Anxa2,Anxa7,Ptgfrn,Anxa6,Anxa4
CL:39109	Intermediate filament protein	0.0094	Krt31,Krt33a,Krt84,Krt18,Krt34,Krt33b,Krt35,Krt36,Krt32,Tgm3
CL:18052	S-100/ICaBP type calcium binding domain, and Annexin	0.0196	S100a4,Anxa11,Anxa1,S100a11,Anxa3,Anxa2,Anxa7,Anxa6
CL:23123	Carbon metabolism, and Pyruvate metabolism	0.027	G6pdx,Pgam1,Mdh2,Sdha,Tkt,Aco2,Pgk2,Pdha1,Pck2,Gpi1,Pepd,Pgd,Idh1,Aco1
CL:21623	mixed, incl. membrane coat, and retromer complex	0.0308	Ap2b1,Snx3,Ehd3,Vps35,Snx2,Picalm,Dnm3,Dnm1,Cltb,Rab4b,Ehd2,Cltc,Clta,Tpd52l2,Ap2a1
CL:39112	Intermediate filament protein	0.0308	Krt31,Krt33a,Krt84,Krt18,Krt34,Krt33b,Krt35,Krt32,Tgm3
CL:14217	Collagen biosynthesis and modifying enzymes	0.0415	Col6a2,Plod3,P4ha1,Plod1,Leprel2,Pcolce,Colgalt1,Col7a1,Col28a1
CL:39117	Intermediate filament protein	0.0415	Krt31,Krt33a,Krt84,Krt18,Krt34,Krt33b,Krt35,Krt32

Fig. S7. PPI enrichment analysis of the entire DEPs at the distal side of the PDL. The results were generated by STRING v.11.5. *: Matrisome protein-enriched area
